# Identification of an epigenetic prognostic signature for patients with lower‐grade gliomas

**DOI:** 10.1111/cns.13587

**Published:** 2021-01-18

**Authors:** Hai Yu, Duanni Zhang, Minxue Lian

**Affiliations:** ^1^ Department of Neurosurgery The First Affiliated Hospital of Xi'an Jiaotong University Xi'an Shaanxi China; ^2^ Center of Brain Science The First Affiliated Hospital of Xi'an Jiaotong University Xi'an Shaanxi China; ^3^ Shaanxi Provincial People's Hospital Xi'an Shaanxi China

**Keywords:** epigenetic signature, immune suppression, lower‐grade gliomas, overall survival, risk stratification

## Abstract

**Introduction:**

Glioma is the most common malignant primary brain tumor with survival outcome for patients with lower‐grade gliomas (LGGs) being quite variable. Epigenetic modifications in LGGs appear tightly linked to patient clinical outcomes but are not commonly used as clinical tools.

**Aims:**

We aimed to derive an epigenetic enzyme gene signature for LGGs that would allow for improved clinical risk stratification.

**Results:**

The study employed transcriptomic data of 711 lower‐grade gliomas from three publically available data sets. Based on least absolute shrinkage and selection operator (LASSO) Cox regression analysis, we discovered a 13‐gene epigenetic signature that strongly predicts poor overall survival in LGGs. The robust prediction ability for survival was further verified in two independent validation cohorts. The signature was also significantly associated with malignant molecular signatures including wild‐type IDH, unmethylated MGMT promoter, and non‐codeletion of 1p19q together with linkage to multiple oncogenic pathways. Interestingly, our results showed that immune infiltration of MDSCs together with mRNA expression of immune inhibition biomarkers was also positively correlated with the epigenetic signature. Lastly, we confirmed the oncogenic role of SMYD2 in glioma tumor cells in functional assays.

**Conclusions:**

We report a novel epigenetic gene signature that harbors robust survival prediction value for LGG patients that is tightly linked to activation of multiple oncogenic pathways.

## Graphical abstract

We identified an epigenetic enzyme gene signature in LGG (lower‐grade glioma) patients that harbors high predictive value of patient's outcome. In addition, the analyses showed that high‐risk signature is tightly associated with immune escape of LGG tumor cells. Our finding presents a new prediction model that provides a potentially better choice for clinical assessment of LGG patients.

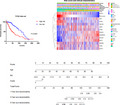

## 1 INTRODUCTION

Glioma is the most common form of malignant primary brain tumor. WHO grading from 2016 classifies gliomas into four grades (I–IV) according to their histopathological signatures with astrocytomas, oligodendrogliomas, and mixed oligoastrocytomas being recognized as lower‐grade gliomas (LGGs).^1^, ^2^ Importantly, the survival of LGG patients ranges widely when subgrouped by histological signature. The most frequent treatment approaches for LGGs are surgical resection combined with chemoradiotherapy.^3^ However, tumor recurrence and malignant progression inevitably occur following treatment, largely owing to the high invasiveness of residual tumor cells and the surgically unapproachable location.^3^ Moreover, in some cases, LGGs may rapidly progress to high‐grade glioblastoma multiforme brain (GBM) tumors, significantly affecting quality of life for these patients.

The histopathological classifications of LGGs are widely recognized; however, this feature alone does not satisfactorily predict patient survival. On this basis, clinical decisions are frequently made using genetic and molecular classifications. For example, IDH1/2,^4^ EGFR,^5^ and ATRX^6^ are well‐known indicators for predicting OS in patients. Further evidence also shows that codeletion of 1p/19q with hypermethylation of the MGMT promoter is critical prognostic factor in LGGs.^7^ Current WHO classifications delineate LGGs into three distinct subgroups on the basis of 1p/19q codeletion and IDH mutation.^1^ However, the predictive role of these factors in evaluating patient survival outcomes is inadequate since LGG patients presenting with the same signature often have different outcomes. Thus, more comprehensive studies are warranted to identify better predictive models to improve patient management and outcomes.

Epigenetic alterations are now widely recognized as a cancer hallmark.^8^ The enzymes involved in cancer epigenomic deregulation can be classified into four functional categories: writers, readers, editors, and erasers.^9^ Many epigenetic regulators with oncogenic properties have been intensively investigated in past studies, for example, IDH1 mutations in glioma have been linked with a hypermethylation phenotype which induces global gene expression alterations.^10^, ^11^ Moreover, accumulating evidence shows that epigenetic modifications are tightly linked with the elevated adaptiveness of cancer cells to the harsh tumor microenvironment, together with their increased invasiveness, therapeutic resistance, and recurrence.^12^, ^13^, ^14^ Instructively, studies have also revealed that the cellular plasticity of glioma cells is highly dependent on epigenetic modifications and their intercellular crosstalk within the microenvironment.^15^ However, the predictive efficiency of individual gene modifications remains inadequate, thus limiting the use of this approach in clinical assessments. To improve clinical applications, further investigation of epigenetic enzyme genes is required to construct a more effective integrated predictive model.

The current study focused on deriving an epigenetic gene signature that can predict LGG patient outcomes. Transcriptomic comparisons undertaken to identify deregulated genes were distilled down to a 13‐gene epigenetic signature with verification across three data sets, including the Kamoun, Gravendeel, and TCGA cohorts. The high‐risk signature was strongly linked with worse clinical outcomes for LGG patients. In addition, multiple oncogenic pathways were found to be activated in the high‐risk group with an interestingly association uncovered between the high risk score and immune suppression pathways. Adding to the bioinformatic analyses, we confirmed the oncogenic role of one of the identified genes, SMYD2, using *in vitro* and *in vivo* assays.

## 2 MATERIALS AND METHODS

### 2.1 Ethics

In this study, the usage of cell line and experimental animals (SCID mice) was approved by the Scientific Ethics Committee of Xi'an Jiaotong University, Xi'an, China.

#### 2.1.1 Patients and data sets

A total of 711 lower‐grade gliomas (LGGs) were investigated in this study. mRNA expression data of public data sets were obtained from “Gliovis” (http://gliovis.bioinfo.cnio.es/). ^16^ Clinical and molecular information from TCGA data set (n = 481), Kamoun data set (n = 126), and Gravendeel data set (n = 104) was also collected. In this study, the TCGA data set was used as the discovery set. The other 2 data sets were applied as validation sets. Patient selection criteria: LGG patients with survival less than 1 month, were excluded from this study. We have summarized the clinicopathological characteristics for all patients in Table 1 and Table S1.

**TABLE 1:** Clinicopathological Characteristics and Genetic Alterations of LGG Patients in the TCGA Cohort

Variable	Subgroup	TCGA (N = 481)	
		N	%
Age	<=40	204	42.4%
	>40	221	45.9%
	NA	56	11.6%
Gender	male	235	48.9%
	female	190	39.5%
	NA	56	11.6%
Grade	II	211	43.9%
	III	227	47.2%
	NA	43	8.9%
IDH status	WT	85	17.7%
	mutant	393	81.7%
	NA	3	0.6%
Chr 1p19q status	non‐codel	347	72.1%
	codel	169	35.1%
	NA	0	0.0%
MGMT promoter	unmethylated	83	17.3%
	methylated	398	82.7%
	NA	0	0.0%
TERT status	WT	146	30.4%
	mutant	120	24.9%
	NA	215	44.7%
ATRX status	WT	301	62.6%
	mutant	177	36.8%
	NA	3	0.6%

#### 2.1.2 Data processing and risk score construction

Transcriptomic data from the TCGA, Kamoun, and Gravendeel data sets were systematically analyzed with the aim to identify a gene signature that captures epigenetic modifications of LGG tumor cells, hereafter referred to as deregulated epigenetic enzyme gene (DEEG) signature.

For the discovery phase, differentially expressed genes (DEGs) were derived from the TCGA data set using the “Gene Expression Profiling Interactive Analysis (GEPIA)” (http://gepia.cancer‐pku.cn/). 207 normal brain specimens from the GTEx Portal were used as controls. TCGA and GTEx samples were re‐analyzed (re‐aligned to hg38 genome and expressions are called using RSEM and Kallisto methods) by the same RNA‐seq pipeline. Gene transcripts per million (TPM) data were used for analysis. Genes of |log2FC| >1 and FDR‐adjusted P value <0.01 were identified as differentially expressed genes. A total of 5745 DEGs were identified and applied against the next screening step of 212 epigenetics enzyme genes to identify a total of 57 deregulated epigenetic enzyme genes (DEEGs). Further analysis using LASSO Cox regression revealed 13 of the 57 selected DEEGs were found to be powerful prognostic LGG biomarkers. The coefficients of 13 DEEGs were used to construct a risk score (RS) model and to assess the predictive accuracy of the risk score; time‐dependent ROC curve analyses were performed.

### 2.2 Bioinformatic analysis

GO (Gene ontology) and Kyoto Encyclopedia of Genes and Genomes (KEGG) analyses were carried out by using a well‐known online software (https://david‐d.ncifcrf.gov/). To visualize the potential links between the DEEGs, we constructed the protein‐protein network using the STRING database. MCODE method in Cytoscape was used to identify clusters among the DEEGs. Gene set enrichment analysis (GSEA) was performed using a well‐known online tool (http://software.broadinstitute.org/gsea/index.jsp). During the analysis, risk score was regarded as a phenotype.

### 2.3 Analyses of immune signature

The 782 immune metagenes were obtained from previous study.^17^ The metagenes contain 28 cell subpopulations. The single‐sample gene set enrichment analysis (ssGSEA) was performed to determine the enrichment scores for all the cases. Analysis for the link of gene expression with immune infiltration was based on an online tool “TIMER (Tumor Immune Estimation Resource, https://cistrome.shinyapps.io/timer/)”.^18^

### 2.4 Statistics

The statistical analyses were carried out using the R software (version 4.0.0), SPSS (version 22.0), or Prism 6 (GraphPad Software). The patient characteristics were analyzed by Pearson's chi‐squared test. Kolmogorov‐Smirnov tests were used to assess data distribution for normality. The Student t test, one‐way analysis of variance (ANOVA), or nonparametric (Mann‐Whitney) test was used to compare differences between groups. To evaluate the independent prognostic value of each factor, univariate and multivariate Cox regression analyses were carried out. The Kaplan‐Meier (K‐M) analysis was performed to investigate the overall survival of LGG patients. Spearman's correlation coefficient was calculated in correlation analysis. *p* < 0.05 was regarded as statistically significant.

## 3 RESULTS

### 3.1 Construction of a risk score model based on the deregulated epigenetic enzyme genes in LGGs

**FIGURE 1:**
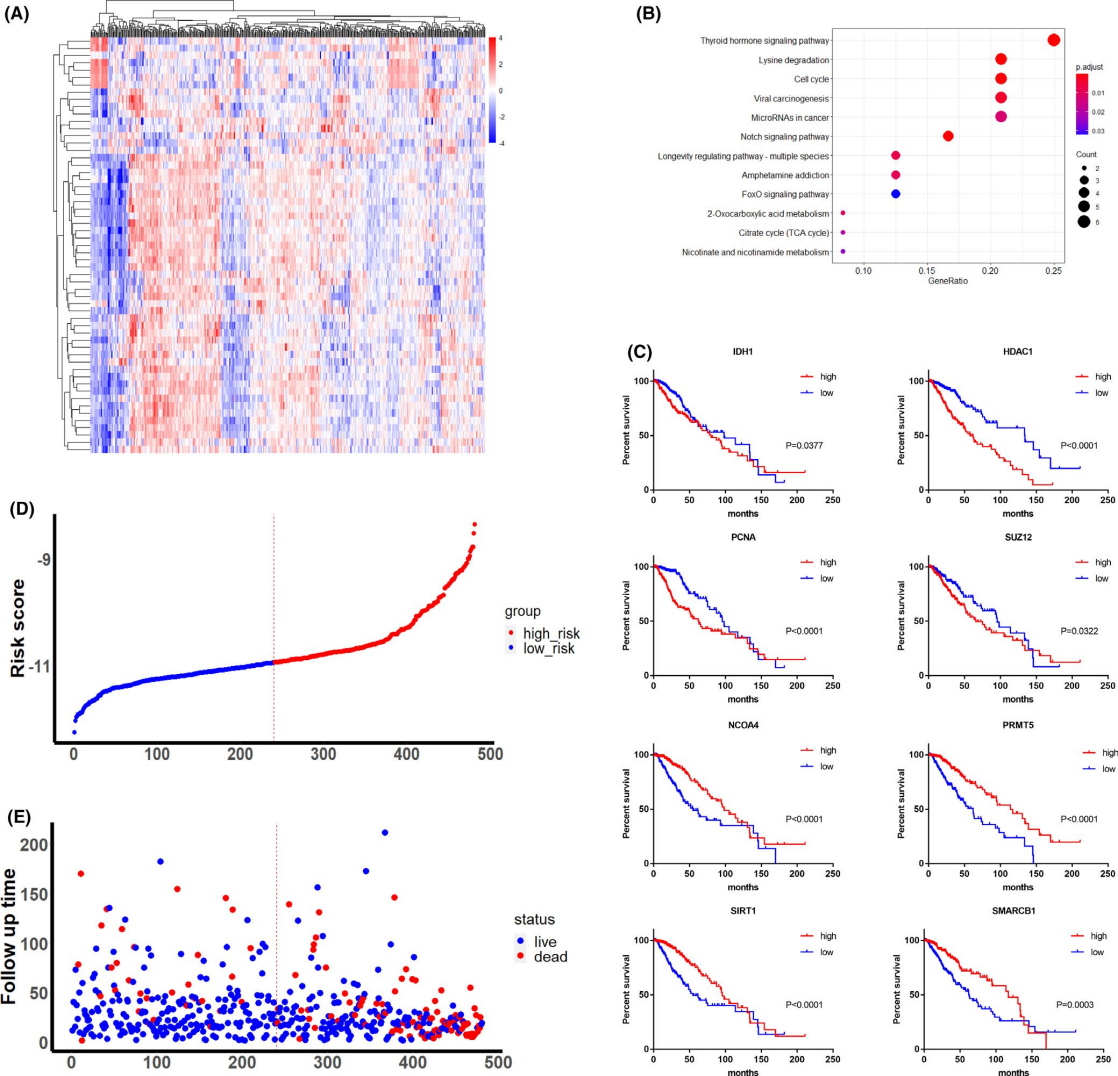
Construction of a risk score model based on the deregulated epigenetic enzyme genes in LGGs. (**A**) Unsupervised clustering result of LGGs based on mRNA expression of 57 deregulated epigenetic enzyme genes. (**B**) Kyoto Encyclopedia of Genes and Genomes (KEGG) analysis of deregulated epigenetic genes from TCGA data set. (**C**) Kaplan‐Meier curve comparing overall survival of LGG patients subgrouped by the expression of IDH1, HDAC1, PCNA, SUZ12, NOCA4, PRMT5, SIRT1, and SMARCB1 in TCGA data set. Log‐rank test. (**D‐E**) Distribution of risk score (**D**), OS, and OS status (**E**) in the TCGA cohort

The lower‐grade glioma patient cohorts used for analysis together with their corresponding studies are shown in Table 1 and Table S1, respectively, with the analysis workflow represented by the flowchart (Figure S1). First, differentially expressed genes (DEGs) were derived from the TCGA data set using the “GEPIA” online tool. To explore the potential roles of epigenetic enzyme genes in LGGs, we next obtained a 212 epigenetic gene list according to a previous study^9^ (Table S2). Compared with normal brain controls, 57 of the 212 epigenetic genes (6 down and 51 upregulated, respectively) were found to be deregulated in LGGs from the TCGA data set (Figure 1A).

To further investigate the DEEGs in the LGG signature, the deregulated epigenetic genes were analyzed using Gene Ontology (GO) and Kyoto Encyclopedia of Genes and Genomes (KEGG) approaches. As expected, GO analysis revealed that chromatin and histone modifications were the top ranked biological processes (Figure S2A). In addition, the pathway enrichment analysis showed that the deregulated genes were tightly linked to a variety of oncogenic pathways, including cell cycle, Notch, FoxO, and metabolite‐associated pathways (Figure 1B), indicating that intra‐tumoral epigenetic status likely plays an important role in LGGs. The protein‐protein interaction (PPI) network of the deregulated genes was further constructed using the STRING database and analyzed by Cytoscape. Consistently, the MCODE analysis identified 3 clusters (cluster score >3) and chromatin and histone modifications were the top enriched pathways (Figure S2B). Collectively, we found that deregulation of epigenetic enzymes is tightly linked to oncogenic progression for LGGs.

Next, we used the LASSO Cox regression analysis to explore the prognostic potential of the 57 identified DEEGs (Figure S2C). The results showed that 13 of the DEEGs, namely IDH1, HDAC1, PHF8, PCNA, SMYD2, ZBTB33, CHD5, NOCA4, CBX7, PRMT5, SUZ12, SIRT1, and SMARCB1, were the most powerful prognostic biomarkers. Among these, lower expression of CHD5, NOCA4, PRMT5, SIRT1, and SMARCB1 was linked to worse outcomes in LGG patients, whereas lower expression of IDH1, HDAC1, PHF8, PCNA, SUZ12, SMYD2, and ZBTB33 significantly contributed to better outcomes (Figure 1C and S2D). Subsequently, based on these 13 biomarkers, we constructed an epigenetic risk score model. The formula for the risk score is presented in Figure S2E. Using the mRNA expression and regression coefficient of each gene, we calculated the risk score for each case in the TCGA data set. The distribution of the risk scores, OS, and OS status in the TCGA cohort is presented in Figure 1D and 1E.

### 3.2 The risk score model harbors robust prediction value for LGG patients

**FIGURE 2:**
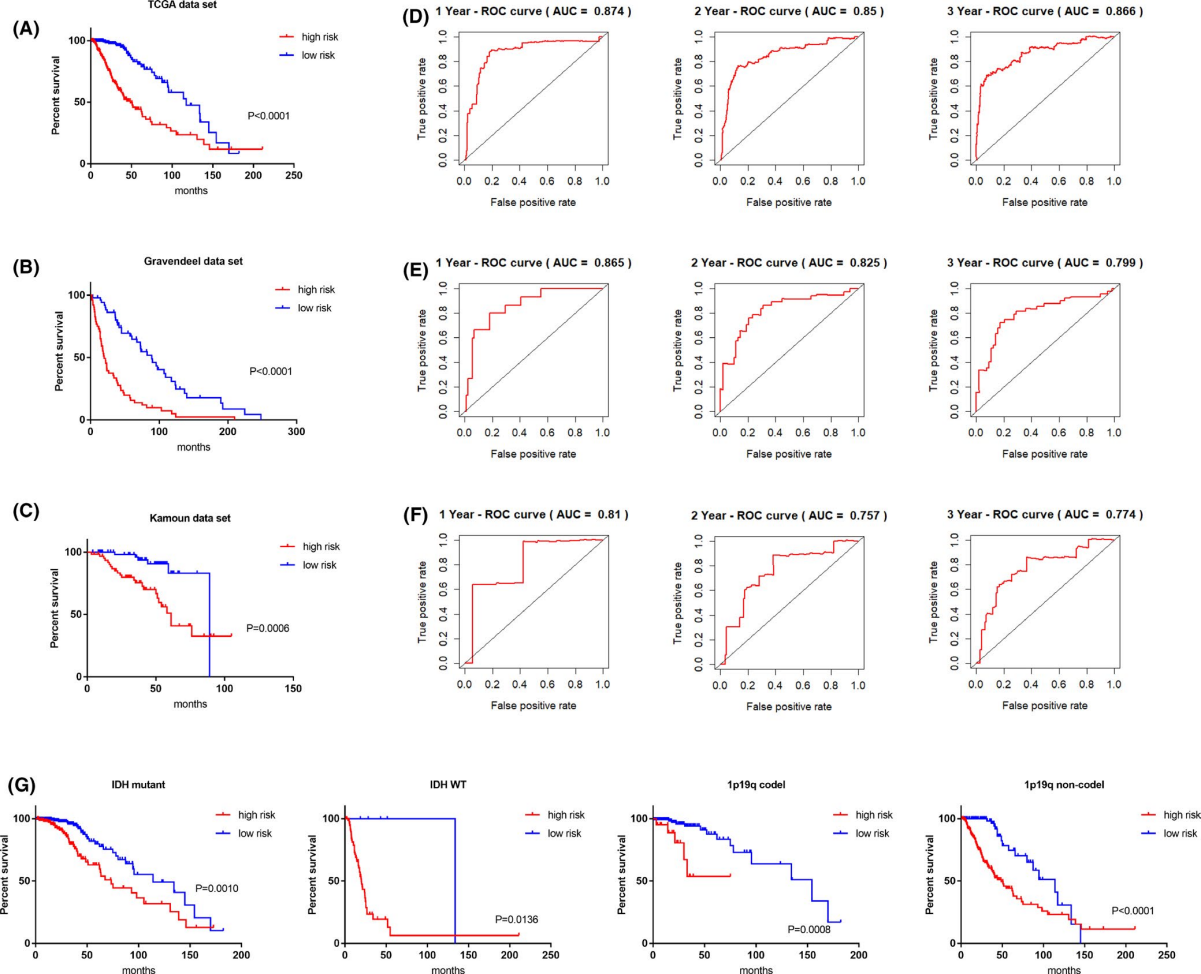
The risk score model harbors robust prediction value for LGG patients. (**A‐C**) Kaplan‐Meier curve comparing overall survival of LGG patients subgrouped by the risk score in the TCGA data set (**A**), Kamoun data set (**B**), and Gravendeel data set (**C**). (**D‐F**) Time‐dependent ROC analysis (1, 2, and 3 years) according to the risk score in the TCGA data set (**D**), Kamoun data set (**E**), and Gravendeel data set (**F**). (**G**) Kaplan‐Meier curve comparing overall survival of LGG patients with high and low risk. The patients with IDH mutation, wild‐type IDH, 1p19q codeletion, and without 1p19q codeletion were analyzed separately

We next evaluated the prognostic value of the epigenetic score by Kaplan‐Meier (K‐M) analyses of patient overall survival using the TCGA data set (Table 1) and then verified the results against the validation data sets (Table S1). Based on the median value of risk scores, the tumor samples were assigned into low‐ or high‐risk groups. As anticipated, patients in the high‐risk group demonstrated significantly poorer outcomes compared with those in the low‐risk group (Figure 2A). Consistently, K‐M survival curves showed that high‐risk patients in the validation data sets had shorter overall survival than low‐risk classified patients (Figure 2B and 2C). Additionally, we evaluated the predictive ability of the risk score using ROC analysis against overall survival at 1, 2, and 3 years (Figure 2D‐F). ROC analyses comparing the performance of the risk signature against of IDH and 1p19q status (Figure S3A‐C) showed that the risk score produced higher AUC values (0.874, 0.85, and 0.866 for 1, 2, and 3 years, respectively). Further analyses of validation data sets confirmed the predictive value of the risk score with AUC values being 0.865, 0.825, and 0.799 (validation data set 1), and 0.81, 0.757, and 0.774 (validation data set 2), respectively. Collectively these analyses highlight the superior performance of the epigenetic signature in predicting patient outcomes.

We further considered our findings in relation to the WHO classification of three different LGG subgroups according to the IDH mutation and 1p/19q codeletion status.^1^ K‐M analyses were performed on patients with high‐ and low‐risk LGGs based on their molecular subtypes. Notably, even within different subgroups we observed that the 13‐gene epigenetic signature was still effective in the predication of patient outcomes (Figure 2G). Moreover, in addition to the molecular subtypes, we applied K‐M analyses against other clinical factors including MGMT promoter status, sex, age, grade, and histology diagnosis. These results showed that the risk score still harbors robust stratification ability for LGG patients (Figure S4A‐K).

### 3.3 The epigenetic gene signature robustly identifies poor molecular signatures in LGGs

**FIGURE 3:**
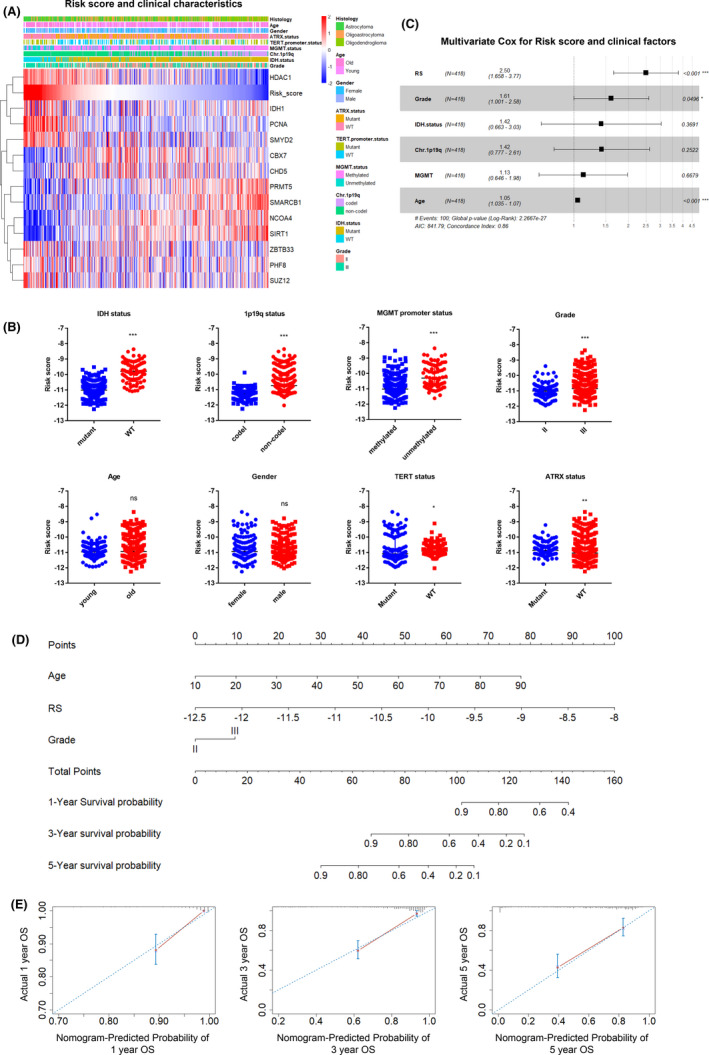
The epigenetic gene signature robustly identifies poor molecular signatures in LGGs. (**A**) Heat map of the risk score and 13 identified genes in the TCGA data set. Grade, IDH mutation status, 1p/19q codeletion status, TERT promoter status, BRAF. V600E status, and MGMT promoter status are annotated in the upper panel. (**B**) Dot plot of the risk score in LGG patients subgrouped by the status of IDH, 1p19q, MGMT promoter, grade, age, gender, TERT status, and ATRX status. (**C**) Forest plot showing the multivariate Cox regression analysis with risk score, age, grade, IDH mutation status, MGMT promoter status, and 1p19q status. RS, risk score, ***p < 0.001, ns, not significant. (**D**) The nomogram for predicting the 1‐year, 3‐year, and 5‐year OS in LGG patients. RS, risk score. (**E**) The calibration plots showed that the nomogram model harbors robust ability in predicting patient outcome

We further investigated the link between the risk score and the aforementioned clinical factors in LGGs. Clinicopathological and genetic alterations, including age, sex, grade, IDH mutation status, MGMT promoter status, Chr. 1p19q status, TERT mutation status, and ATXR mutation status, are summarized in Table 1. As shown in Figure 3A, higher risk scores demonstrated stronger associative trends with older age, higher tumor grade, wild‐type (WT) IDH, 1p19q non‐codeletion (non‐codel), and non‐methylation of the MGMT promoter. We subgrouped the LGGs based on these signatures and compared the risk score value between each group. The result further validated our observations (Figure 3B). In addition, analyses using Pearson's chi‐squared test (Table 2) demonstrated that higher risk score was significantly linked to higher tumor grades (grade III, *p* < 0.001), WT IDH (*p* < 0.001), non‐codel status of 1p19q (*p* < 0.001), non‐methylation status of the MGMT promoter (*p* < 0.001), and WT status of TERT (*p* < 0.001). However, no significant differences were identified with sex or age.

**TABLE 2:** Clinicopathological Correlations of the Epigenetic Enzyme Gene Signature in the TCGA Cohort

Variable	N	Epigenetic risk score		p value
		High risk score	Low risk score	
Age	<=40	96	108	0.456
	>40	112	109	
Gender	male	112	123	0.557
	female	96	94	
Grade	II	86	125	0.001
	III	127	100	
IDH status	WT	80	5	<0.001
	mutant	160	233	
Chr 1p19q status	non‐codel	220	103	<0.001
	codel	21	137	
MGMT promoter	unmethylated	70	13	<0.001
	methylated	171	227	
TERT status	WT	81	65	0.035
	mutant	51	69	
ATRX status	WT	134	167	0.001
	mutant	106	71	

Next, we performed univariate and multivariate Cox regression analyses to determine if the prediction ability of the epigenetic signature was independent of other factors. The univariate analysis showed that age, grade (II vs. III), IDH mutation status (mutant vs. WT), 1p19q status (codel vs. non‐codel), MGMT promoter methylation status (methylated vs. unmethylated), and the risk score all derived significant hazard ratios (HR) in the TCGA data set (Table S3). Factors giving significant results were applied in a multivariate analysis where the risk score was found to be prognostic independently of other clinical factors for patient overall survival (HR =2.50, 95% CI =1.658–3.77) (Figure 3C).

### 3.4 Nomogram model construction based on the epigenetic signature

To further assess the clinical application of the epigenetic model, we constructed a nomogram, incorporating risk score and clinicopathological parameters such as age and grade (Figure 3D). The nomogram generated to predict the 1‐year, 3‐year, and 5‐year overall survival in the TCGA data set demonstrated good calibration and discrimination ability for predicting LGG patient outcomes (Figure 3E). Indeed, the high C‐index (0.86) indicated that our model exhibited very good performance in this assessment.

### 3.5 Pathway enrichment analysis of the epigenetic gene signature

**FIGURE 4:**
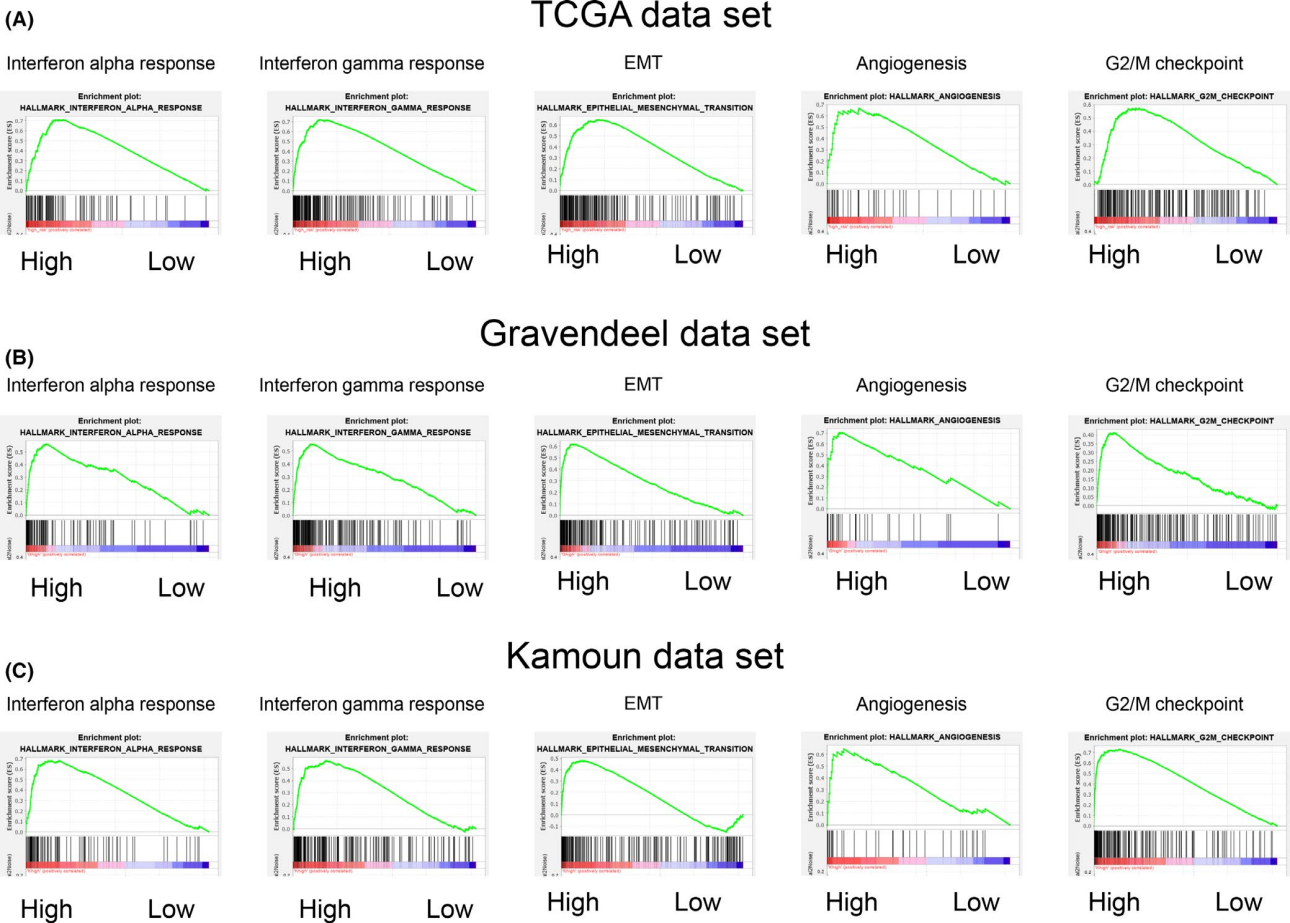
Pathway enrichment analysis of the epigenetic gene signature. (**A‐C**) Gene set enrichment analysis (GSEA) of LGG patient samples from TCGA (**A**), Kamoun (**B**), and Gravendeel data sets (**C**) subgrouped by the risk score

We next sought to explore the molecular basis of the epigenetic signature using gene set enrichment analysis (GSEA) to better identify the underlying oncogenic pathways involved. Here we employed the TCGA data set using the risk score as a phenotype label (high risk vs. low risk) (Table S4). This analysis showed that the interferon alpha/gamma response, the epithelial‐mesenchymal transition (EMT), the G2/M checkpoint, angiogenesis, the TNF‐α signaling via NFκB, and the E2F targets were significantly activated in the high‐risk group (Figure 4A and S5). The same pathway enrichments were observed in the validation data sets (Figure 4B, 4C and S5), collectively indicating that critical pathways linked with tumor invasion and infiltration such as epithelial‐mesenchymal transition (EMT)^19^ and angiogenesis^20^ are activated in high‐risk LGGs. Moreover, the therapeutic response‐associated pathways were found to be highly enriched in the high‐risk group, including G2/M checkpoint^21^ and E2F target^22^ pathways. Interestingly, we also found that the pathways strongly associated with immune response were significantly activated in the high‐risk group (interferon response, inflammation response, and TNF‐α signaling, respectively). The latter finding suggests that epigenetic status could contribute to intra‐tumoral immune heterogeneity, thereby contributing a critical role in the cellular interactions between tumor cells and immune cells.

### 3.6 The risk score model is closely associated with immune suppression in LGGs

**FIGURE 5:**
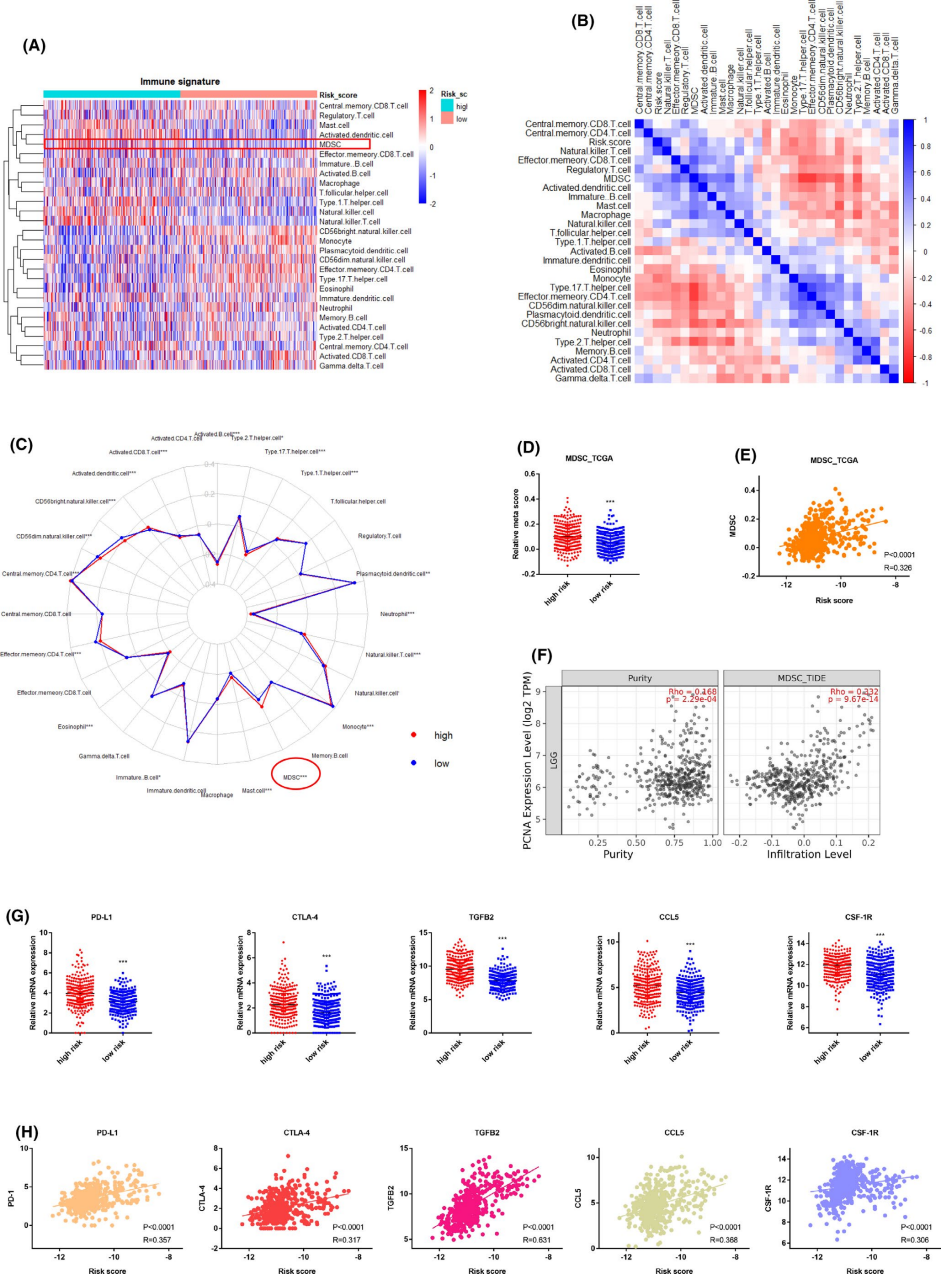
The risk score model is closely associated with immune suppression in LGGs. (**A**) Heat map of different immune cell populations in LGGs from TCGA data set. The ssGSEA scores was applied. (**B**) The correlation matrix showing the correlation between 28 immune cell subpopulations and the risk score. (**C**) Radar map showing the immune infiltration difference between high‐ and low‐risk subgroups. (**D**) Dot plot showing the meta_score for MDSCs in high‐ or low‐risk groups for TCGA data set. (**E**) Correlation analysis of the risk score with meta_score of MDSCs in TCGA data set. (**F**) Infiltration correlation analysis of PCNA with MDSC, using “Timer” web tool. (**G**) Dot plot showing the expression of PD‐L1, CTLA‐4, TGFβ2, CCL5, and CSF‐1R in high‐ or low‐risk groups. (**H**) Correlation analysis of the risk score with mRNA expression of PD‐L1, CTLA‐4, TGFβ2, CCL5, and CSF‐1R.***p < 0.001

Given the above findings, we sought to explore the link between the risk score and immune status in LGGs. We first performed ssGSEA to assess the infiltration scores of different immune cell subpopulations (Figure 5A). Interestingly among the 28 cell subpopulations, myeloid‐derived suppressor cells (MDSCs), natural killer (NK) T cells, and active dendritic cells showed significantly higher correlation with the high risk score (Figure 5B and 5C). Previous studies have showed that the majority of infiltrating immune cells in glioma are microglia, blood‐derived macrophages, and MDSCs.^23^ In addition, MDSCs is a well‐known cell population tightly linked with immune suppression in glioma, inducing T‐cell apoptosis, Treg cell activation, and functional impairment of NK cells and active dendritic cells.^24^, ^25^ Thus, we performed further focused investigations on MDSCs.

We observed a significantly higher meta‐score for MDSCs in the high‐risk group (Figure 5D). Moreover, correlation analyses between risk score and the meta‐score further confirmed this observation (Figure 5E). Similar correlation result was observed in the validation data sets (Figure S6A and S6B). Infiltration correlation analysis using the “TIMER” online tool showed that PCNA was the main contributor to MDSC enrichment in LGGs (Figure 5F). In addition to the infiltration of immune suppressive cells in LGGs, we also investigated the relationship between the risk score and the expression of immune suppressive biomarkers. Notably, the results revealed that the epigenetic signature was tightly linked with mRNA expression levels of PD‐L1, PD‐1, CTLA‐4,^26^ TGFβ1/2/3,^27^ CCL5,^28^ IL‐10,^29^ CSF‐1, and CSF‐1R,^30^ highlighting an elevated immune suppressive microenvironment in these tumor samples (Figure 5G and S6C). In addition, correlation analyses identified that the epigenetic score was positively associated with these immune suppression biomarkers (Figure 5H and S6D). Collectively, these data propose a strong link between the risk score model and tumor immune suppression.

### 3.7 Functional validation of oncogenic role of SMYD2 in glioma

We next investigated the oncogenic role of SMYD2, one of the 13 identified biomarkers where analysis of the TCGA data set showed significantly elevated expression of in the high‐risk group (Figure S7A).

**FIGURE 6:**
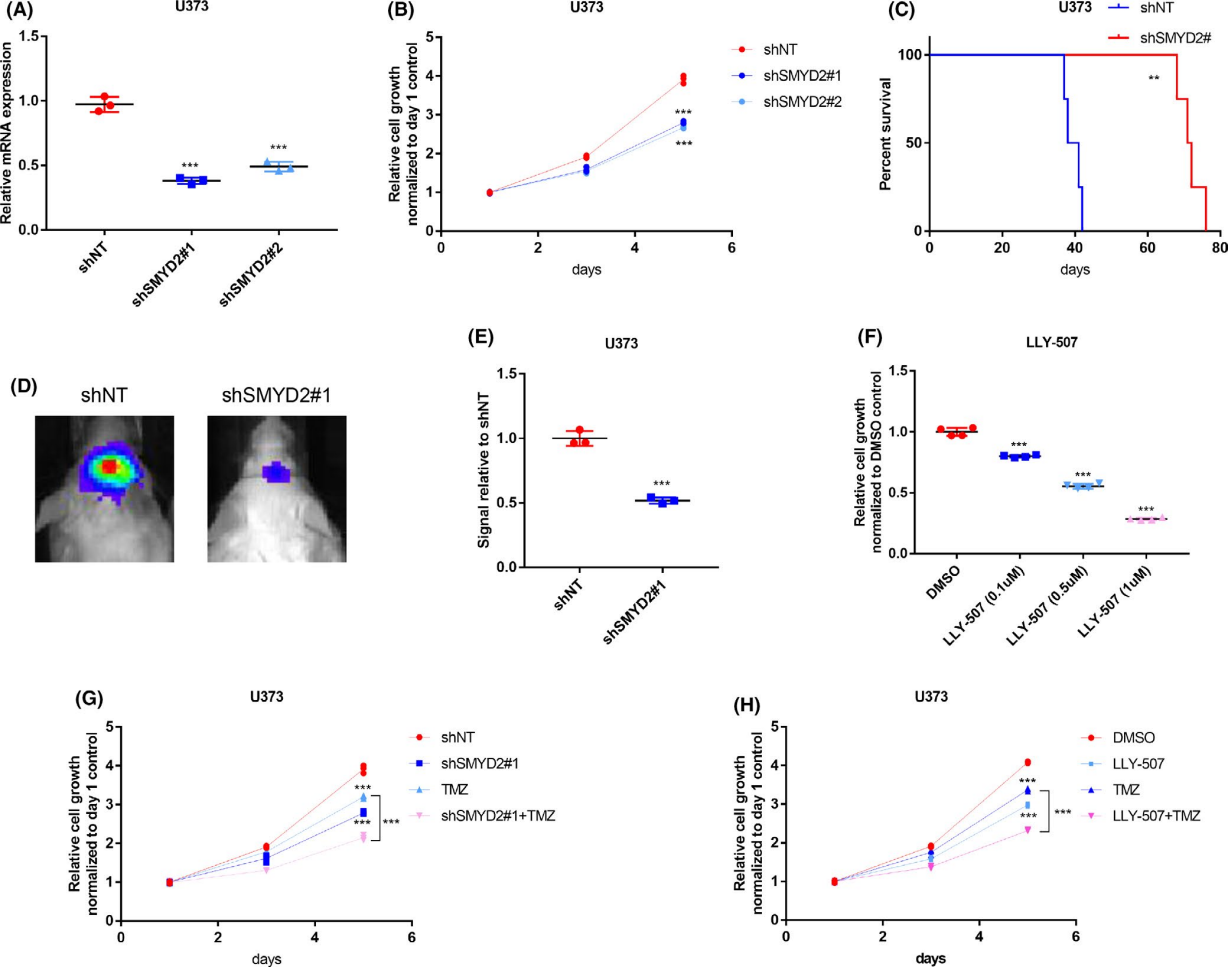
Functional validation of SMYD2 oncogenic role in glioma. (**A**) qRT‐PCR analysis of SMYD2 mRNA expression in U373 glioma cells treated with NT‐shRNA or SMYD2‐shRNA. GAPDH served as control. (**B**) *In vitro* cell viability assay of U373 glioma cells treated with either NT‐shRNA or SMYD2‐shRNA. (**C**) Kaplan‐Meier curve comparing overall survival of mice intracranially injected with U373 glioma cells pre‐treated with either NT‐shRNA or SMYD2‐shRNA. Log‐rank test. (**D**) Representative bioluminescence images of mice injected with glioma cells pre‐infected with either NT‐shRNA or SMYD2‐shRNA on day 25. (**E**) Signal quantification for Figure 6D. (**F**) *In vitro* cell viability assay of U373 glioma cells treated with either DMSO or LLY‐507 (SMYD2 inhibitor) on day 3. (**G**) *In vitro* cell viability assay of U373 glioma cells treated with/without TMZ after pre‐treatment with either NT‐shRNA or SMYD2‐shRNA. (**H**) *In vitro* cell viability assay of U373 glioma cells treated with/without TMZ after pre‐treatment with either DMSO or LLY‐507 (SMYD2 inhibitor). **p < 0.01, ***p < 0.001

To directly investigate the functional role of SMYD2, we infected U373 glioma cells with lentiviruses expressing either non‐targeting control (shNT) or two independent shRNAs targeting SMYD2 (SMYD2#1 and #2, respectively). The silencing efficacy was evaluated by qRT‐PCR, indicating significant reductions in SMYD2 mRNA expression with a relatively higher silencing efficacy of shSMYD2#1 compared with shSMYD2#2 (Figure 6A). Assessment of the *in vitro* growth of the U373 glioma cells demonstrated that their proliferative capacity was attenuated after SMYD2 silencing in comparison to the shNT‐treated cells (Figure 6B). As further *in vivo* validation, shNT or shSMYD2‐bearing U373 glioma tumor cells were intracranially injected into the brains of SCID mice. The outcome revealed a significantly prolonged survival time in the shSMYD2 group (Figure 6C) with serial bioluminescent imaging (Figure 6D and 6E) confirming that SMYD2 silencing attenuated the tumor forming ability and progression of xenografted glioma cells. As independent confirmation of these data, experiments utilizing the SMYD2 inhibitor, LLY‐507, demonstrated that targeting SMYD2 was effective in inhibiting the proliferation of U373 glioma cells (Figure 6F). Remarkably, shSMYD2 treatment significantly reduced the expression of three other epigenetic regulators in the 13‐gene risk set, namely HDAC1, PCNA, and SUZ12, implying that SMYD2 expression contributes to other elements of the epigenetic risk score (Figure S7B). Collectively these data indicate that SMYD2 is essential to glioma tumor cell proliferation *in vitro* and tumor formation *in vivo*.

Finally, given the importance of temozolomide (TMZ) in the therapeutic management of glioma following surgical resection, we investigated whether SMYD2 confers TMZ resistance to glioma. Glioma cells were treated with TMZ alone and in combination with shRNA and inhibitor approaches to target SMYD2. Instructively, both shRNA silencing of SMYD2 (Figure 6G) and LLY‐507 inhibitor treatment (Figure 6H) potentiated the effects of TMZ of cell growth when compared with control group. Together these data suggest that SMYD2 influences the resistance of glioma cells to TMZ with implications for the chemoradiotherapy of glioma.

## 4 DISCUSSION

Several studies have already described significant links between the distinct molecular subtypes and poor patient prognosis in glioma.^4^, ^5^, ^6^, ^7^ Nevertheless, the clinical application of these factors, especially for individual patients, has thus far not been satisfactory. Recent evidence shows that the epigenetic modifications in tumor cells play a critical role in the progression of many cancers including gliomas.^31^ Thus, in this study, we focused on the exploration and construction of an epigenetic signature to provide an alternative assessment model that could be used clinically.

We used the resources of the TCGA as our discovery cohort and screened for known epigenetic enzyme genes that are aberrantly expressed in LGGs. Consequently, we identified a 13 deregulated epigenetic enzyme gene (DEEG) signature which after validation showed high predictive value for patient outcomes. Moreover, the high risk score was tightly linked with malignant LGG features including wild‐type IDH status, unmethylated MGMT promoter, and non‐codeletion status of 1p19q. Additionally, pathway enrichment analysis showed that the epigenetic signature was strongly associated with a variety of oncogenic pathways including immunity as further described below.

Collectively, our risk score model defined a variety of oncogenic genes that are involved in multiple epigenetic modification processes, depicting a general map of epigenetic alternations for LGGs and enabling higher prediction value for patient outcome. Of the 13 selected biomarkers, higher expression of IDH1, HDAC1, PCNA, SUZ12, PHF8, SMYD2, and ZBTB33 was significantly correlated with worse outcome in LGG patients. Many of these enzymes are strongly implicated in LGG tumorigenesis. For instance, the mutation status of IDH1 and less commonly IDH2 has been well recognized as crucial factors for classification and prognostic prediction in LGGs. The mutation of IDH1 or IDH2 leads to the elevated production of the oncometabolite 2‐hydroxyglutarate, which is thought to induce significant epigenetic alterations and promote the initiation and progression of tumor cells.^10^ For HDAC1, a well‐known histone deacetylase, a number of studies have validated its crucial role in tumor progression.^32^, ^33^, ^34^ One recent study also found that PCNA is strongly associated with the stemness and radio‐resistance in glioma tumor cells.^35^ SUZ12, a critical factor in the PRC2 complex, has also been found to promote therapeutic resistance and intra‐tumoral heterogeneity in GBM.^31^, ^36^ Another study found that ZBTB33 is highly expressed in gliomas and tightly linked with proliferation, invasion, and EMT phenotype.^37^ For PHF8 and SYMD2, even though reports have confirmed their oncogenic roles in other cancer types, little is known about the function of these biomarkers in glioma.

On this basis, we chose to further explore the oncogenic role of SMYD2 in glioma. SMYD2 functions as a lysine methyltransferase and has been previously shown to promote proliferation, epithelial‐mesenchymal transition, and invasion of a variety of cancer cell types.^38^, ^39^, ^40^ In addition, one study found that positive expression of SMYD2 was associated with poor prognosis in patients with hepatocellular carcinoma.^41^ The interaction between SMYD2 and EZH2,^39^ a critical factor in glioma progression, further highlights its potential oncogenic role in LGGs. Here we showed through shRNA silencing and inhibitor approaches that SMYD2 is essential for cell proliferation, tumor formation, and TMZ resistance in glioma tumor cells. However, further mechanistic investigations are warranted for a deeper understanding of SMYD2 functions in this context.

One highly interesting observation concerned the strong enrichment of a variety of immune‐associated pathways in the high‐risk group, indicating a potential link between our risk score model and the immune microenvironment in LGGs. To verify our hypothesis, the ssGSEA analysis and expression correlation analysis were further carried out to explore the link of the epigenetic gene signature with immune status in LGGs. The analyses confirmed the link between higher infiltration of MDSCs and the high risk score. Accumulating evidence from the literature reveals a critical role of MDSCs in immune suppression, as well as their prominent role in tumor invasion, and therapeutic resistance.^42^ Thus, the samples with a high risk score are likely to be more immune suppressive. Furthermore, the high risk scores were also closely associated with elevated mRNA expression for immune suppression biomarkers (TGFβ1/2/3, etc.) and the immune checkpoint biomarkers (PD‐1, PD‐L1, and CTLA‐4). Previous studies have reported that TGFβ is capable of slowing down the anti‐tumor activation of T cells and lead to a more immunosuppressive microenvironment.^27^, ^43^ In addition, the binding of PD‐L1 (tumor cells) with PD‐1 (T cells) can lead to exhaustion of activated T cells.^44^, ^45^ Therefore, tumor cells create an immunosuppressive environment via this interaction to escape from immune system attack.^45^ Recent evidence has also shown that PD‐L1 engagement can also inhibit phagocytosis of tumor‐associated macrophages.^46^ Additionally, cytotoxic T lymphocyte–associated protein‐4 (CTLA‐4), another crucial immune checkpoint marker expressed on T cells, is capable of decreasing the activation of T helper and effector cells while stimulating Tregs.^26^, ^47^ Thus, it is likely that the higher infiltration of multiple immune cells in high‐risk group promotes malignant LGG progression through a variety of mechanisms including the increased involvement of MDSCs and the effects of immune checkpoint and suppression markers which disable the activation of active immune cells.

The limitations of this study should be noted in particular, the reliance on data derived from bioinformatic analyses. While we did perform preliminary investigations on the role of SMYD2, many key elements of the study need further verification. For example, the elevated infiltration of immune cells such as MDSCs needs to be further verified. Our mouse model experiments used SCID mice which lack an immune component, so other approaches are needed to better define this aspect. In addition, the clinical application of the risk score model in immune therapy assessment may need further verification. Previous studies (Tumor Immune Dysfunction and Exclusion, TIDE)^48^ have established an online data set for immune response prediction; however, its application in glioma is limited due to the lack of appropriate clinical samples from glioma patients undergoing immunotherapy. A comprehensive data set of glioma patients with detailed immune therapy response record is therefore necessary for this verification.

Additionally, the risk score may also guide the current standard post‐surgical treatments, including chemotherapy and irradiation treatment. High correlations between the risk score and unmethylated MGMT promoter indicate that patients with high risk scores may display an unfavorable response to temozolomide (TMZ), the most frequently used chemotherapy drug for glioma.^49^ Similarly, the enrichment of multiple oncogenic pathways, including G2/M checkpoint,^21^ E2F targets,^22^ and EMT,^19^ is strong indicator for an irradiation‐resistant phenotype. These findings therefore imply utility for the risk score model in therapeutic response assessment. However, further analyses of patients with detailed therapeutic records are necessary for the validation of our hypothesis.

In conclusion, we focused on understanding of the epigenetic enzyme deregulation in LGGs. Through a comprehensive analysis, a novel, 13‐gene epigenetic signature has been identified and validated. This signature harbors robust risk stratification ability and facilitates a better prediction of overall outcome for LGG patients compared with existing criteria. Our findings suggest that epigenetic deregulations are tightly linked to oncogenic process alterations, and the signature can be applied as a prediction tool in clinical assessment. Furthermore, the risk score may help guide and assess immunotherapy.

## CONFLICTS OF INTEREST

We claim that no conflicts of interest exist in the submission and the manuscript has been approved by all authors.

## Supporting information

Fig S1

Fig S2

Fig S3

Fig S4

Fig S5

Fig S6

Fig S7

Table S1

Table S2

Table S3

Table S4

Supplementary Material

## Data Availability

The data used to support the findings of this study are available from the corresponding author upon request.
