# Supplementary figures and images for: Identification of an epigenetic prognostic signature for patients with lower‐grade gliomas
Source: CNS Neurosci Ther. 2021 Jan 18;27(4):470–83. doi: 10.1111/cns.13587 (PMC7941239; doi:10.1111/cns.13587)

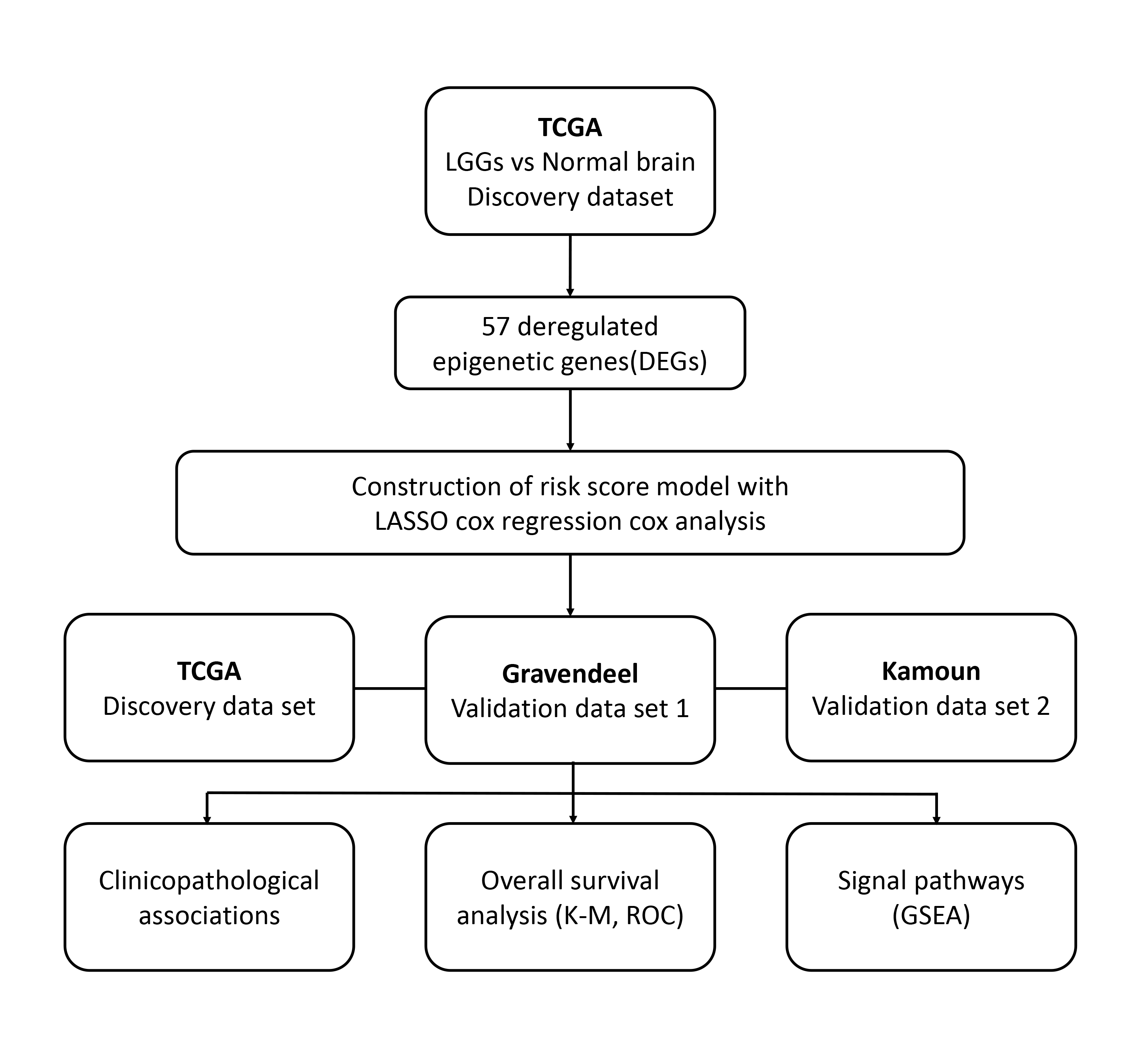

Supplement: Supplementary file 1 — Fig S1 [file CNS-27-470-s010.tif]

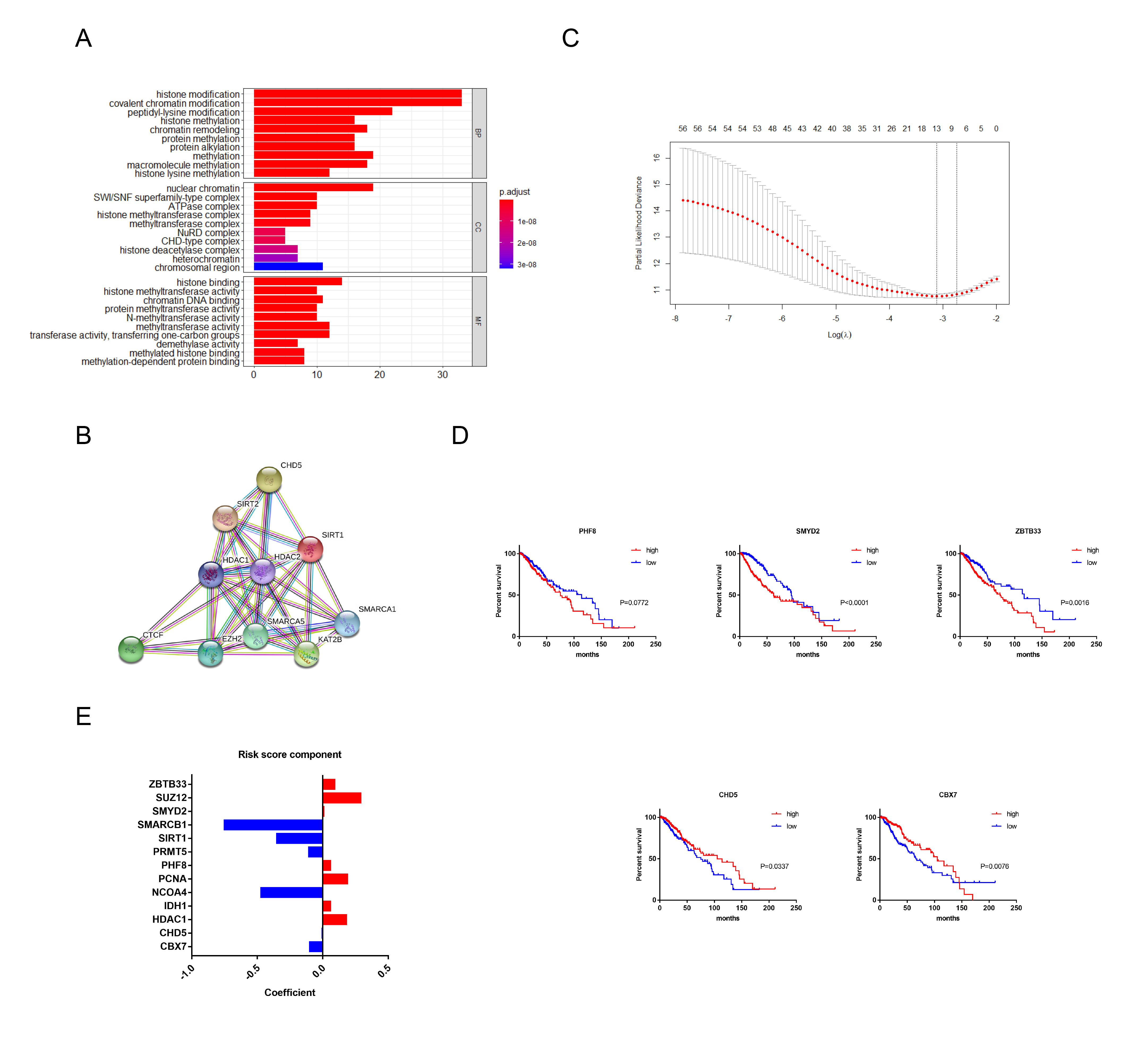

Supplement: Supplementary file 2 — Fig S2 [file CNS-27-470-s002.tif]

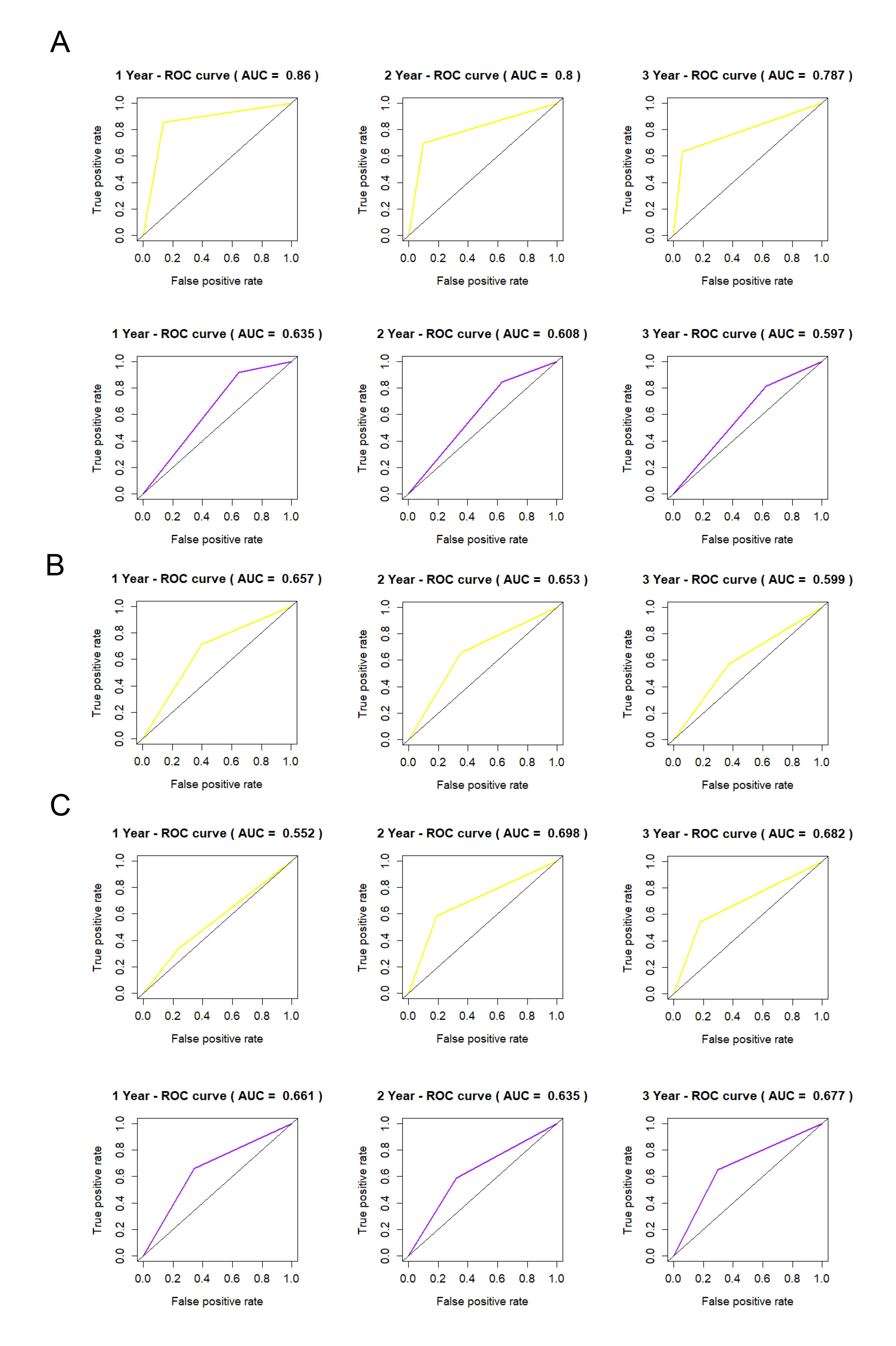

Supplement: Supplementary file 3 — Fig S3 [file CNS-27-470-s012.tif]

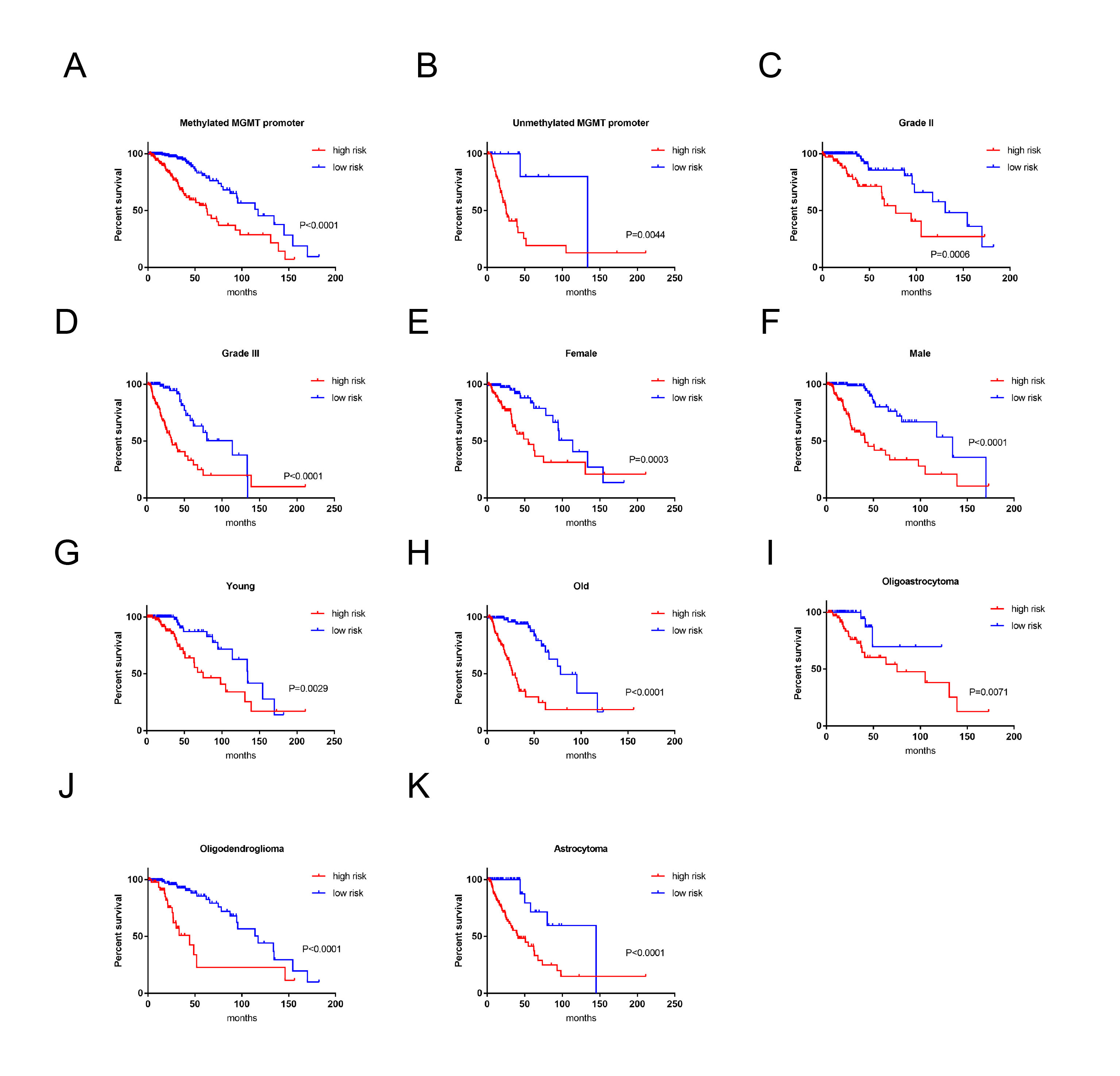

Supplement: Supplementary file 4 — Fig S4 [file CNS-27-470-s005.tif]

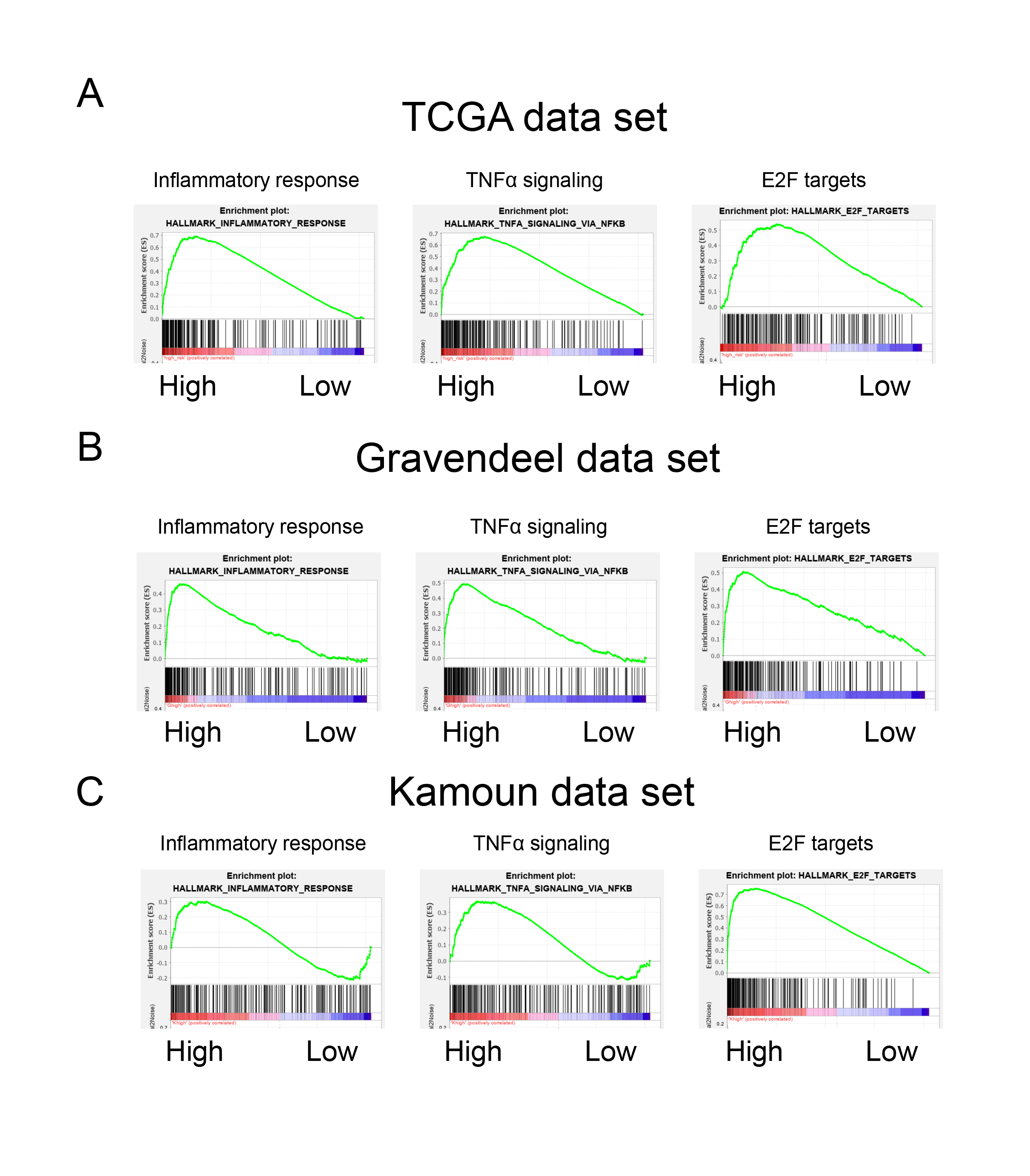

Supplement: Supplementary file 5 — Fig S5 [file CNS-27-470-s007.tif]

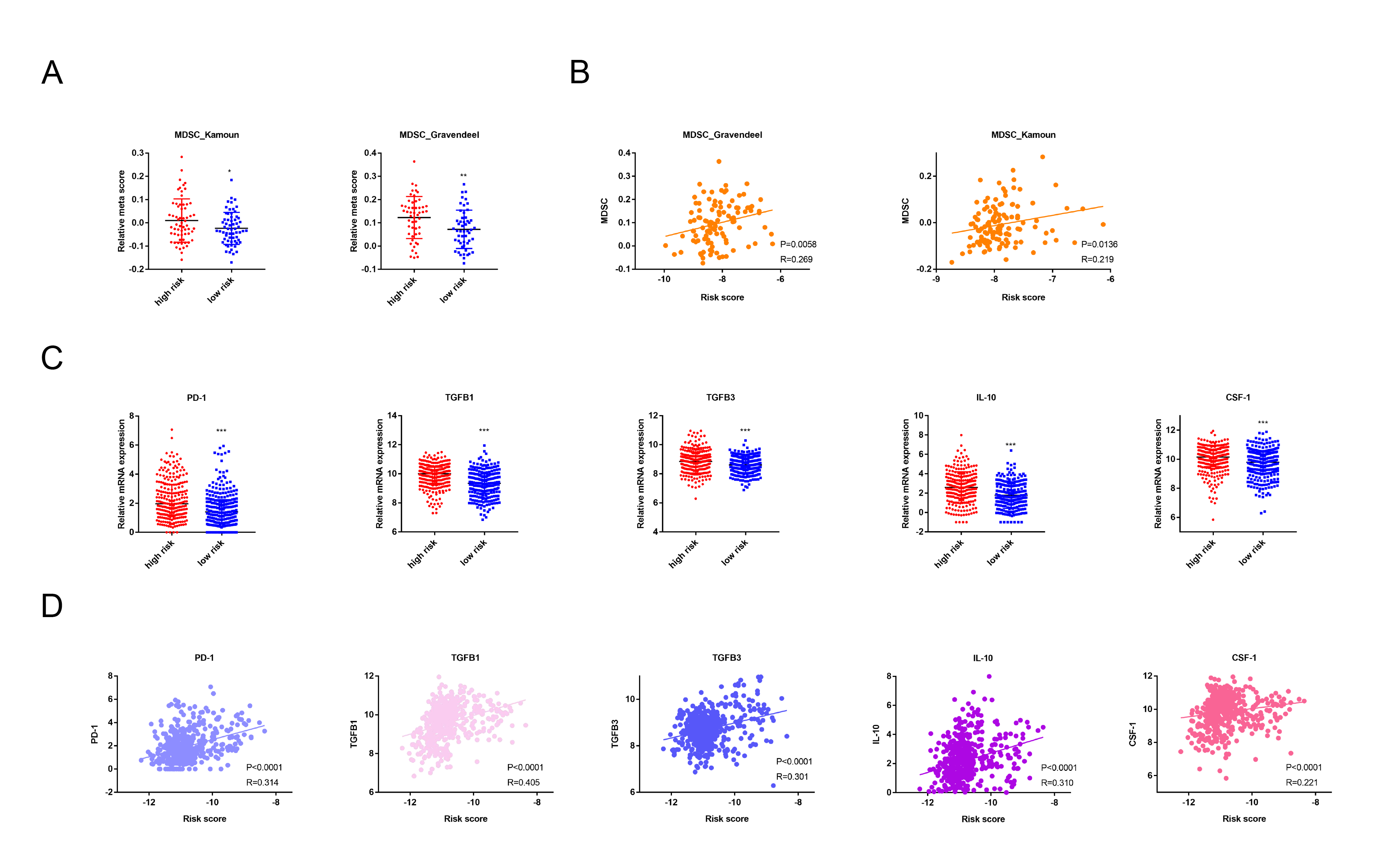

Supplement: Supplementary file 6 — Fig S6 [file CNS-27-470-s004.tif]

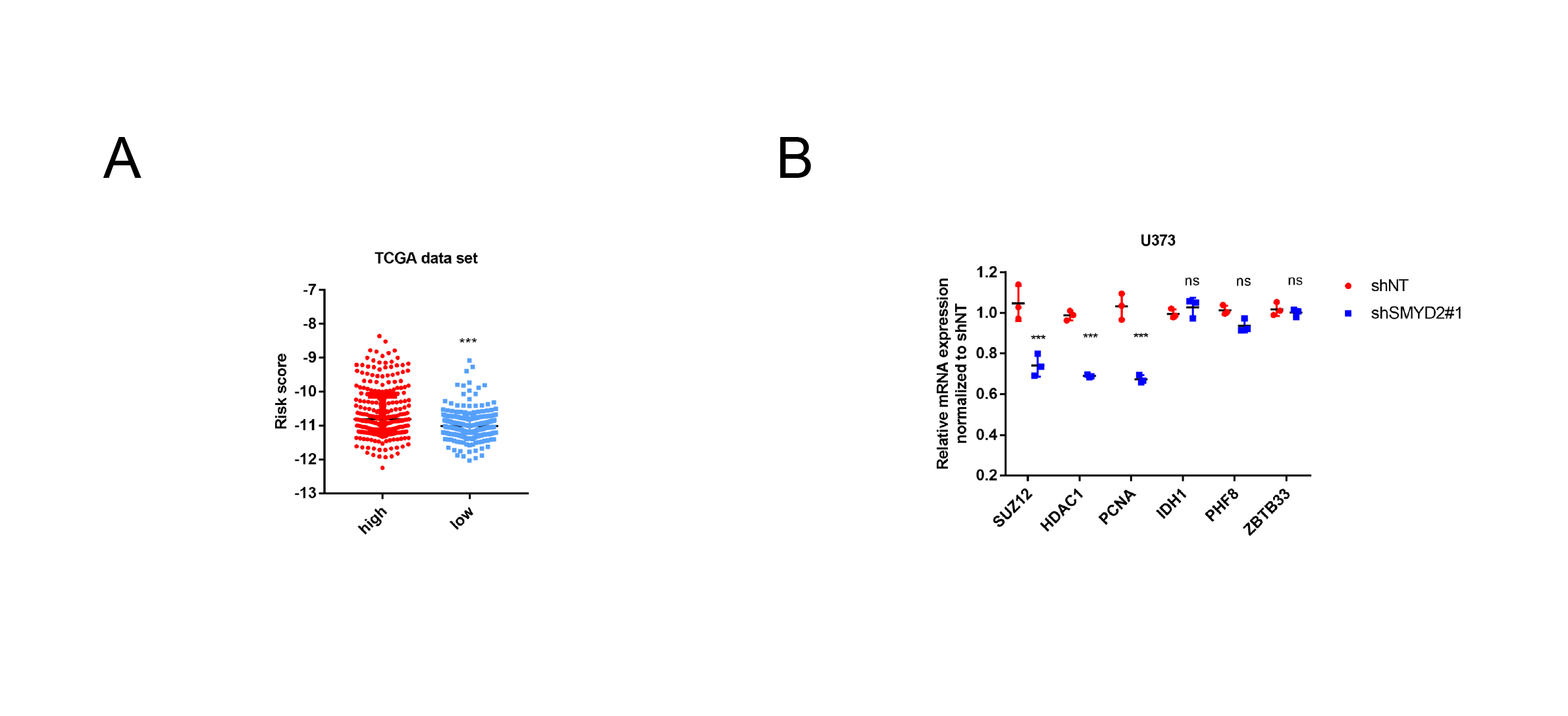

Supplement: Supplementary file 7 — Fig S7 [file CNS-27-470-s008.tif]
