# Supplementary material for: Identification of an epigenetic prognostic signature for patients with lower‐grade gliomas
Source: CNS Neurosci Ther. 2021 Jan 18;27(4):470–83. doi: 10.1111/cns.13587 (PMC7941239; doi:10.1111/cns.13587)
Supplement: Supplementary file 8 — Table S1 [file CNS-27-470-s009.docx]

| **Table S1. Clinicopathological characteristics and genetic alterations of LGG patients in the validation cohorts** | | | | | |
| --- | --- | --- | --- | --- | --- |
| **Variable** | **subgroup** | **Kamoun (N=126)** | | **Gravendeel (N=104)** | |
|  |  | **N** | **%** | **N** | **%** |
| Age | <=40 | 78 | 61.9% | 40 | 38.5% |
|  | >40 | 49 | 38.9% | 64 | 61.5% |
|  | NA | 0 | 0.0% | 0 | 0.0% |
| Gender | male | 73 | 57.9% | 68 | 65.4% |
|  | female | 53 | 42.1% | 36 | 34.6% |
|  | NA | 0 | 0.0% | 0 | 0.0% |
| Grade | II | 39 | 31.0% | 23 | 22.1% |
|  | III | 87 | 69.0% | 81 | 77.9% |
|  | NA | 0 | 0.0% | 0 | 0.0% |
| IDH status | WT | 29 | 23.0% | 46 | 44.2% |
|  | mutant | 97 | 77.0% | 38 | 36.5% |
|  | NA | 0 | 0.0% | 20 | 19.2% |
| Chr 1p19q status | non-codel | 44 | 34.9% | - | - |
|  | codel | 82 | 65.1% | - | - |
|  | NA | 0 | 0.0% | - | - |
