# Supplementary material for: Identification of an epigenetic prognostic signature for patients with lower‐grade gliomas
Source: CNS Neurosci Ther. 2021 Jan 18;27(4):470–83. doi: 10.1111/cns.13587 (PMC7941239; doi:10.1111/cns.13587)
Supplement: Supplementary file 9 — Table S2 [file CNS-27-470-s011.docx]

| **Table S2. List of 212 epigenetic enzyme genes** | | | | | |
| --- | --- | --- | --- | --- | --- |
| Gene Symbol | | | | | |
| DNMT1 | KAT5 | PRDM14 | KDM3A | ZMYM2 | ZMYND8 |
| DNMT3A | MYST3 | PRDM15 | KDM3B | ZMYM3 | NIPBL |
| DNMT3B | MYST4 | PRDM16 | KDM4A | ZMYM4 | SUZ12 |
| DNMT3L | MYST2 | PRMT1 | KDM4B | ZMYM5 | EED |
| MBD1 | MYST1 | PRMT2 | KDM4C | ZMYM6 | CTCF |
| MBD2 | EP300 | PRMT3 | KDM4D | UTY | SMARCA1 |
| MBD3 | CREBBP | PRMT5 | KDM5A | TAF1 | SMARCB1 |
| MBD4 | NCOA1 | PRMT6 | KDM5B | TAF3 | SMARCA4 |
| MECP2 | NCOA2 | PRMT7 | KDM5C | BRD4 | SMARCA5 |
| IDH1 | NCOA3 | PRMT8 | KDM5D | BRD8 | SRCAP |
| IDH2 | NCOA4 | PRMT10 | KDM6A | RAG2 | ATRX |
| TET1 | NCOA5 | SET | KDM6B | BPTF | BTAF1 |
| TET2 | NCOA6 | SETBP1 | JMJD1C | PHF2 | HELLS |
| TET3 | NCOA7 | SETD1A | JMJD6 | PHF6 | TTF2 |
| ZBTB33 | GTF3C1 | SETD1B | SIRT1 | PHF8 | ERCC6 |
| ZBTB4 | CLOCK | SETD2 | SIRT2 | MECOM | INO80 |
| ZBTB38 | ASH1L | SETD3 | SIRT3 | CBX5 | RAD54L |
| PCNA | EHMT1 | SETD4 | SIRT4 | CBX7 |  |
| UHRF1 | EHMT2 | SETD5 | SIRT5 | WHSC1 |  |
| AICDA | EZH1 | SETD6 | SIRT6 | GLYATL1 |  |
| ALKBH1 | EZH2 | SETD7 | SIRT7 | TP53BP1 |  |
| ALKBH3 | MLL | SETD8 | EYA1 | DOT1L |  |
| APOBEC1 | MLL2 | C5orf35 | EYA2 | MORF4L1 |  |
| FTO | MLL3 | SETDB1 | EYA3 | BRPF1 |  |
| TDG | MLL4 | SETDB2 | EYA4 | ADNP |  |
| MGMT | MLL5 | SETMAR | SMEK1 | ATXN7 |  |
| HDAC1 | NSD1 | SMYD1 | SMEK2 | DHX30 |  |
| HDAC2 | PRDM1 | SMYD2 | SMEK3P | EP400 |  |
| HDAC3 | PRDM2 | SMYD3 | DUSP1 | MGA |  |
| HDAC4 | PRDM4 | SMYD4 | CHD1 | GABRG1 |  |
| HDAC5 | PRDM5 | SMYD5 | CHD2 | CARM1 |  |
| HDAC6 | PRDM6 | SUV39H1 | CHD3 | GATAD2A |  |
| HDAC7 | PRDM7 | SUV39H2 | CHD4 | GATAD2B |  |
| HDAC8 | PRDM8 | SUV420H1 | CHD5 | HCFC1 |  |
| HDAC9 | PRDM9 | SUV420H2 | CHD6 | HCFC2 |  |
| HDAC10 | PRDM10 | KDM1A | CHD7 | TRRAP |  |
| HDAC11 | PRDM11 | KDM1B | CHD8 | SMC1A |  |
| KAT2A | PRDM12 | KDM2A | CHD9 | SMCHD1 |  |
| KAT2B | PRDM13 | KDM2B | ZMYM1 | POGZ |  |
