# Supplementary material for: Identification of an epigenetic prognostic signature for patients with lower‐grade gliomas
Source: CNS Neurosci Ther. 2021 Jan 18;27(4):470–83. doi: 10.1111/cns.13587 (PMC7941239; doi:10.1111/cns.13587)
Supplement: Supplementary file 10 — Table S3 [file CNS-27-470-s001.docx]

| **Table S3. Univariate Cox regression analysis for clinical clinicopathological and genetic alterations** | | | | |
| --- | --- | --- | --- | --- |
| **Variable** | **P value** | **HR** | **95% CI** | |
|  |  |  | **lower** | **upper** |
| Grade (II vs III) | 1.2999E-06 | 2.917 | 1.891 | 4.501 |
| IDH (mutant vs WT) | 6.8022E-20 | 6.816 | 4.515 | 10.291 |
| 1p19q (codel vs non-codel) | 0.00018173 | 2.656 | 1.593 | 4.43 |
| MGMT (methylated vs unmethylated) | 1.1771E-05 | 2.612 | 1.7 | 4.014 |
| Age | 1.6545E-12 | 1.059 | 1.042 | 1.076 |
| Gender (male vs female) | 0.97609817 | 0.994 | 0.67 | 1.476 |
