# Supplementary material for: Identification of an epigenetic prognostic signature for patients with lower‐grade gliomas
Source: CNS Neurosci Ther. 2021 Jan 18;27(4):470–83. doi: 10.1111/cns.13587 (PMC7941239; doi:10.1111/cns.13587)
Supplement: Supplementary file 11 — Table S4 [file CNS-27-470-s006.docx]

| **Table S4 GSEA analyses based on the risk score subgroup** | | | | |
| --- | --- | --- | --- | --- |
| **TCGA data set** | | | | |
| NAME | ES | NES | NOM p-val | FDR q-val |
| INTERFERON_GAMMA_RESPONSE | 0.7214 | 2.3556 | 0 | 0 |
| ALLOGRAFT_REJECTION | 0.7072 | 2.3121 | 0 | 0 |
| INFLAMMATORY_RESPONSE | 0.6917 | 2.2717 | 0 | 0 |
| TNFA_SIGNALING_VIA_NFKB | 0.6725 | 2.2065 | 0 | 0 |
| INTERFERON_ALPHA_RESPONSE | 0.7114 | 2.1954 | 0 | 0 |
| IL6_JAK_STAT3_SIGNALING | 0.6951 | 2.1326 | 0 | 0 |
| EPITHELIAL_MESENCHYMAL_TRANSITION | 0.6500 | 2.1174 | 0 | 0 |
| IL2_STAT5_SIGNALING | 0.6040 | 1.9853 | 0 | 0 |
| COMPLEMENT | 0.6003 | 1.9639 | 0 | 0 |
| COAGULATION | 0.6064 | 1.9293 | 0 | 0 |
| APOPTOSIS | 0.5905 | 1.9072 | 0 | 0 |
| G2M_CHECKPOINT | 0.5722 | 1.8675 | 0 | 0.00E+00 |
| ANGIOGENESIS | 0.6668 | 1.8005 | 0.002587 | 2.31E-04 |
| KRAS_SIGNALING_UP | 0.5452 | 1.7962 | 0 | 0.000215 |
| E2F_TARGETS | 0.5391 | 1.7674 | 0 | 0.0002 |
| HYPOXIA | 0.5179 | 1.6943 | 0 | 0.000751 |
| ESTROGEN_RESPONSE_LATE | 0.4666 | 1.5294 | 0 | 0.007799 |
| P53_PATHWAY | 0.4450 | 1.4523 | 0.001026 | 0.0207 |
| MITOTIC_SPINDLE | 0.4388 | 1.4420 | 0 | 0.0225 |
| GLYCOLYSIS | 0.4377 | 1.4311 | 0.003132 | 0.024343 |
| **Gravendeel data set** | | | | |
| NAME | ES | NES | NOM p-val | FDR q-val |
| INTERFERON_GAMMA_RESPONSE | 0.5543 | 4.3618 | 0 | 0 |
| EPITHELIAL_MESENCHYMAL_TRANSITION | 0.6178 | 4.2131 | 0 | 0 |
| INFLAMMATORY_RESPONSE | 0.4604 | 4.0210 | 0 | 0 |
| TNFA_SIGNALING_VIA_NFKB | 0.4949 | 3.9500 | 0 | 0 |
| E2F_TARGETS | 0.5082 | 3.7223 | 0 | 0 |
| INTERFERON_ALPHA_RESPONSE | 0.5622 | 3.5834 | 0 | 0 |
| COMPLEMENT | 0.4178 | 3.5643 | 0 | 0 |
| ANGIOGENESIS | 0.7066 | 3.5567 | 0 | 0 |
| G2M_CHECKPOINT | 0.4111 | 3.1319 | 0 | 0 |
| COAGULATION | 0.4338 | 2.8435 | 0 | 0 |
| ALLOGRAFT_REJECTION | 0.3486 | 2.8286 | 0 | 0 |
| HYPOXIA | 0.4083 | 2.8148 | 0 | 0 |
| GLYCOLYSIS | 0.3619 | 2.7948 | 0 | 0 |
| IL6_JAK_STAT3_SIGNALING | 0.4063 | 2.7242 | 0 | 0 |
| APOPTOSIS | 0.3671 | 2.4924 | 0 | 0.00E+00 |
| KRAS_SIGNALING_UP | 0.3087 | 2.1298 | 0 | 0.00E+00 |
| ESTROGEN_RESPONSE_LATE | 0.2712 | 2.0171 | 0 | 1.29E-03 |
| MTORC1_SIGNALING | 0.2654 | 1.8822 | 0 | 4.46E-03 |
| IL2_STAT5_SIGNALING | 0.2641 | 1.8323 | 0 | 4.22E-03 |
| MITOTIC_SPINDLE | 0.2540 | 1.7681 | 0 | 6.20E-03 |
| **Kamoun data set** | | | | |
| NAME | ES | NES | NOM p-val | FDR q-val |
| E2F_TARGETS | 0.7519 | 3.1407 | 0 | 0 |
| G2M_CHECKPOINT | 0.7298 | 3.0345 | 0 | 0 |
| INTERFERON_ALPHA_RESPONSE | 0.6815 | 2.5433 | 0 | 0 |
| INTERFERON_GAMMA_RESPONSE | 0.5664 | 2.3337 | 0 | 0 |
| MITOTIC_SPINDLE | 0.5014 | 2.0960 | 0 | 0 |
| ANGIOGENESIS | 0.6464 | 2.0512 | 0 | 0 |
| MYC_TARGETS_V1 | 0.4902 | 2.0396 | 0 | 0 |
| EPITHELIAL_MESENCHYMAL_TRANSITION | 0.4811 | 1.9798 | 0 | 0 |
| IL6_JAK_STAT3_SIGNALING | 0.4824 | 1.7798 | 0 | 0.000685 |
| MYC_TARGETS_V2 | 0.5138 | 1.7701 | 0 | 0.000617 |
| ALLOGRAFT_REJECTION | 0.4243 | 1.7552 | 0 | 0.000728 |
| DNA_REPAIR | 0.4217 | 1.6631 | 0 | 0.003076 |
| WNT_BETA_CATENIN_SIGNALING | 0.5132 | 1.6150 | 0.019022 | 0.004612 |
| APOPTOSIS | 0.3955 | 1.5860 | 0 | 0.006092 |
| NOTCH_SIGNALING | 0.5143 | 1.5501 | 0.023438 | 0.007975 |
| TNFA_SIGNALING_VIA_NFKB | 0.3682 | 1.5283 | 0.005236 | 0.010012 |
| TGF_BETA_SIGNALING | 0.4354 | 1.4749 | 0.017143 | 0.015188 |
| COAGULATION | 0.3417 | 1.3691 | 0.008621 | 0.038259 |
| INFLAMMATORY_RESPONSE | 0.3025 | 1.2603 | 0.026178 | 0.084828 |
| IL2_STAT5_SIGNALING | 0.2773 | 1.1637 | 0.067633 | 1.70E-01 |
