# Supplementary material for: Identification of an epigenetic prognostic signature for patients with lower‐grade gliomas
Source: CNS Neurosci Ther. 2021 Jan 18;27(4):470–83. doi: 10.1111/cns.13587 (PMC7941239; doi:10.1111/cns.13587)
Supplement: Supplementary file 12 — Supplementary Material [file CNS-27-470-s003.docx]

**Supplementary materials and methods**

**Reagents and Antibodies**

Following reagents were used: DMEM-F12, Fetal bovine serum (FBS), Penicillin-Streptomycin, Trypsin-EDTA (Thermo scientific). Anti-β-Actin (3700, Cell signaling, WB), anti-SMYD2 (9734, Cell signaling, WB), anti-rabbit IgG-Horseradish peroxidase (NA934V), anti-mouse IgG-Horseradish peroxidase (NXA931) (GE Healthcare).

***In Vitro* Cell Cultures**

Glioma cell line U373 is provided by Xi’an Jiaotong University. Glioma cells were cultivated in DMEM/F12 medium containing 10% FBS supplement (vol%), 1% Penicillin-Streptomycin solution and the cultural medium was changed every 3 to 4 days.

**RNA Isolation and Quantitative Real-Time PCR**

The detailed method for qRT-PCR was described in previous study(1). The primer sequences used in this study include the following:

SMYD2 (forward TACTGCAATGTGGAGTGTCAGA; reverse ACAGTCTCCGAGGGATTCCAG)

GAPDH (forward: GGAGCGAGATCCCTCCAAAAT; reverse: GGCTGTTGTCATACTTCTCATGG**)**

IDH1 (forward TGTGGTAGAGATGCAAGGAGA; reverse TTGGTGACTTGGTCGTTGGTG)

SUZ12 (forward: AGGCTGACCACGAGCTTTTC; reverse: GGTGCTATGAGATTCCGAGTTC**)**

PCNA (forward CCTGCTGGGATATTAGCTCCA; reverse CAGCGGTAGGTGTCGAAGC)

HDAC1 (forward CTACTACGACGGGGATGTTGG; reverse GAGTCATGCGGATTCGGTGAG)

PHF8 (forward: GTGCCGGTGTATTGCCTCT; reverse: CAACACAACTGCCATGAAACC**)**

ZBTB33 (forward TGCTGAACTCCTTGAATGAGC; reverse CGGAATTTTCGGTCTTCCACAA)

**Western Blot**

The detailed method for western blot (WB) was described in previous study(1). The membrane was blocked with 5% Blotting Grade Blocker Non-Fat Dry Milk (Bio-Rad) for 1 hour and then incubated with corresponding primary antibody overnight and next incubated with peroxidase conjugated secondary antibodies for 1 hour. Staining was visualized with Amersham ECL Western Blot System.

**Cell Viability Assay**

Viability of tumor cells was determined using AlamarBlue reagent (Thermo scientific). Cells were seeded at 1,000 cells per well in a 96 well plate, after indicated period of time AlamarBlue reagent was added into each well and 6 hours later fluorescence was measured (Excitation 515-565 nm, Emission 570-610 nm) using Synergy HTX multi-mode reader (BioTek).

***In Vivo* Intracranial Xenograft Tumor Models**

The detailed method for xenograft model was described in previous study(1). 6-8 weeks old SCID mice were used. For *in vivo* bioluminescent imaging, animals were administrated intraperitoneally with 2.5 mg/100ul solution of XenoLight D-luciferin (PerkinElmer) and anesthetized with isoflurane for the imaging analysis. IVIS 100 imaging system (PerkinElmer).

**Lentivirus Production and Transduction**

The detailed method for lentivirus infection was described in previous study(1). Sequence for used shRNA: SMYD2#1: CCGGGCTGTGAAGGAGTTTGAATCACTCGAGTGATTCAAACTCCTTCACAGCTTTTTG (Sigma, TRCN0000276083); SMYD2#2: CCGGCGATATTTCCTGATGTTGCATCTCGAGATGCAACATCAGGAAATATCGTTTTTG (Sigma, TRCN0000276082).

1. Yu H, Li Z, Wang M. Expression and prognostic role of E2F transcription factors in high-grade glioma. CNS Neurosci Ther. 2020;26(7):741-53.
